# Constructive episodic simulation in dreams

**DOI:** 10.1371/journal.pone.0264574

**Published:** 2022-03-22

**Authors:** Erin J. Wamsley

**Affiliations:** Department of Psychology and Program in Neuroscience, Furman University, Greenville, South Carolina, United States of America; Sapienza University of Rome: Universita degli Studi di Roma La Sapienza, ITALY

## Abstract

Memories of the past help us adaptively respond to similar situations in the future. Originally described by Schacter & Addis in 2007, the “constructive episodic simulation” hypothesis proposes that waking thought combines fragments of various past episodes into imagined simulations of events that may occur in the future. This same framework may be useful for understanding the function of dreaming. N = 48 college students were asked to identify waking life sources for a total of N = 469 dreams. Participants frequently traced dreams to at least one past or future episodic source (53.5% and 25.7% of dreams, respectively). Individual dreams were very often traced to multiple waking sources (43.9% of all dreams with content), with fragments of past memory incorporated into scenarios that anticipated future events. Waking-life dream sources are described in terms of their phenomenology and distribution across time and sleep stage, providing new evidence that dreams not only reflect the past, but also utilize memory in simulating potential futures.

## Episodic future simulation in dreams

Human memory functions not only to help us remember the past, but also to prepare us for the future [1]. This prospective function of memory is thought to be reflected in the content of spontaneous cognition, with a growing interdisciplinary field examining how imagery, thought, and daydreams simulate potential futures [1–7]. According to the “constructive episodic simulation” hypothesis [6], mental stimulations draw on material from our episodic memory stores, reactivating and flexibly recombining fragments of multiple past experiences, and knitting them into novel imagined scenarios that anticipate possible future events. A “core network” of brain regions including medial temporal, medial prefrontal, midline, and parietal regions is engaged both when participants recall the past and when they imagine possible futures, suggesting that these processes rely on a common neural substrate [3, 4, 8, 9]. This network strongly overlaps with the so-called “default network”, which becomes maximally active during stimulus-free rest periods and is associated with spontaneous thought and imagery that often includes remembering the past and/or imagining the future [8, 10–12]. Together, these and other observations have led to the proposal that a critical function of the brain is to use past memories in constructing simulations of possible futures, thus enhancing one’s preparedness for the future [1].

The current study applies the concept of constructive episodic simulation to the content and function of dreaming. Our hypotheses were motivated, first, by evidence that sleep and dreaming support the consolidation of recent memory. Across diverse forms memory, sleep following encoding results in improved memory performance after a delay [13, 14]. The primary candidate mechanism for this effect is an iterative reactivation and strengthening of recently formed memory networks in the sleeping brain [13, 15–17].

Yet critically, sleep not only quantitatively strengthens memory, but also qualitatively transforms past memory traces in a way that may help us respond to similar events in the future. The literature is replete with examples of sleep’s role in extracting commonalities across items [18–20] or reorganizing information to solve future problems [21, 22]. Sleep has also been proposed to especially benefit memory for information needed in the near future [[23] although see Wamsley et al. [24]], and intentions to perform specific actions in the future [25, 26]. Sleep, therefore, is thought to not only to strengthen past memory, but to reorganize memory in a way that benefits future performance.

Emerging evidence suggests that dreaming is related to these putative memory functions of sleep. First, while dreams are rarely an exact replication of any particular past experience, they very often incorporate isolated elements drawn from recent episodic memories [27–31]. While it remains unclear exactly how the particular memory fragments appearing in dreams are selected, we know this is not a simple function of the amount of *time* we spend on an activity during the day–Experiences that take up a large amount of our time, such as reading or working on a computer, appear in dreams less often than shorter-duration but potentially more meaningful categories of events, including social interactions and experiences rated as being significant, novel, or concerning [32, 33]. This suggests that dreams may preferentially incorporate experiences that are in some way personally important to the dreamer. In support of this view, emotional intensity is reportedly higher for daytime experiences that are incorporated into dreams, relative to those which do not appear in dreams [32, 33]. Together, these observations suggest that dreams incorporate fragments of episodic memory in a non-random fashion, perhaps preferentially including episodic memory content of special significance or salience to the dreamer.

Importantly, when recent experiences appear in dreams, even in fragmentary form, subsequent memory for those experiences is improved. Across a number of recent studies, it has been demonstrated that after completing a laboratory-introduced learning task, participants who report task-related dreams show greater improvements in performance after sleep, relative to participants who do not dream about the learning task [34–37].

But again, these learning-related dreams include only fragments of content drawn from the learning experience, rather than replaying the experience in full. For example, after being informed they would be playing a skiing arcade game the following day, one participant in a past study dreamed of a movie trailer they had recently seen for a snowboarding-themed comedy set at a ski resort, rather than dreaming of the skiing game itself [30]. Thus, dreaming of recently learned information appears to benefit its consolidation, even though these dreams are an amalgam of episodic memory fragments, rather than a strict “replay” of what has been learned.

Taken together, these observations suggest that sleep and dreaming do not function merely to replay and strengthen past memory in its original form. Instead, as in waking cognition, the novel combination of episodic memory details in dreams might provide a vehicle for the predictive simulation of possible future events, an emergent process occurring when fragments of multiple different future-relevant memories are simultaneously reactivated in the sleeping brain. The current study was designed to test three predictions derived from this central idea:

1. *First*, *we hypothesized that participants would commonly identify future events as the source of dream content recalled from all sleep stages*. Although the proportion of dreams associated with future events is unknown, analyses of dream content have long suggested that dreams do at least sometimes reference anticipated episodes. For example, students dream of upcoming exams [38], patients dream of upcoming surgeries [39], pregnant women dream of their future baby [40], and athletes dream of approaching competitions [41]. Experimentally introduced future events can induce dreams in the laboratory environment as well. In one study, we informed participants they would be playing a downhill skiing arcade game the following morning. Dream reports contained thought and imagery of game play, despite the fact that participants had not yet seen the task [30].
2. *Second*, *in line with the constructive episodic simulation hypothesis*, *we expected that dreams about the future would be constructed from fragments of past memory*. Thus, we anticipated that dreams would frequently be traced to multiple different past and future waking sources, with fragments of waking experience combined into novel scenarios relevant to anticipated events in participants’ personal futures. However, we did not expect dream “simulations” of future events to be realistic. Dreams are almost never a faithful reiteration of an entire past episodic memory. Instead, they incorporate unbound fragments of past experience, woven into a scenario that may bear little resemblance to the memory a participant identifies as its “source” [27, 28]. Similarly, we expected that dreams “about the future” in the current study would consist of future-relevant elements drawn from past episodic and semantic memories, but incorporated into dreams that may not realistically depict the future event.
3. *Third*, *we expected to find that even dreams without a future source would combine fragments from multiple past memories*. Sleep and dreaming likely serve a multiplicity of functions [42], and as such, we did not expect all dreams to related to an upcoming future event. The simultaneous reactivation of multiple past experiences could serve other functions as well, such as to extract commonalities across related episodes [16, 28, 43] or to integrate new information into established semantic networks [44, 45].

## Methods

To test these hypotheses, we conducted a laboratory-based sleep study during which each of 48 participants were awakened to provide up to 13 dream reports across a full night of sleep. During a morning interview, participants listened to recordings of the previous night’s reports, and were queried about potential past and future memory sources of each recalled dream.

### Participants

Participants were N = 48 undergraduate students, aged 18–25 (mean 20.3yrs ±1.4SD; 60% female). This was a convenience sample, in which participants were recruited through advertisement from the student populations of Furman University in Greenville, SC (n = 39) and Boston College in Boston, MA (n = 9). This research was approved by the institutional review boards of both Furman University, Greenville, SC, and Beth Israel Deaconess Medical Center, Boston, MA.

By self-report, participants indicated that they were free of medications that could interfere with normal sleep patterns (including antidepressants, stimulants, hypnotics, opiates, and anticholinergics), had never been diagnosed with a sleep or mental disorder, and were fluent in English. Participants were asked to keep a regular sleep schedule for the 3 nights prior to the study (defined as no bedtimes before 10pm or after 2am), confirmed by sleep log. Finally, participants were asked to refrain from drugs or alcohol during the day prior to the study, and to refrain from consuming caffeine after 10 am on the day of the study.

Other data from this same study were reported in a 2016 paper on the effect of test expectation on memory consolidation [24]. However, the data, research questions, analyses and conclusions described here are non-overlapping with those addressed in Wamsley et al. 2016. The study was originally designed to test both sets of hypotheses, and the hypotheses described in the current paper were formulated prior to data collection.

### Procedures

Upon arrival at the laboratory, participants provided written informed consent prior to completing a series of questionnaires inquiring about demographic information and sleep history, the Epworth Sleepiness Scale (a measure of trait sleepiness [46]), and the Stanford Sleepiness Scale (a measure of state sleepiness [47]). As a part of the research questions addressed in Wamsley et al., 2016, all participants were trained on a 3D-style spatial navigation learning task prior to sleep, and were retested this task the following morning. Details of this training procedure are described in Wamsley et al. [24].

#### Polysomnography

Prior to sleep, participants were wired for polysomnographic recording, with EEG recording sites including F3, F4, C3, C4, O1, and O2, each referenced to the contralateral mastoid. Additional electrodes acquired electromyography (EMG; muscle tone) and electrooculography (EOG; eye movement). Signals were recorded at 400Hz using a Grass-Telefactor AURA amplifier (© Grass Technologies). Impedance was kept below 10kOhms. Following data collection, polysomnographic recordings were scored for sleep stage following the criteria established by the American Academy of Sleep Medicine [48].

#### Collection of dream reports

Following an experimental bedtime of ≈11:00pm, participants were intermittently awoken to provide verbal reports of their subjective experiences. Report collection procedures followed the methods established in our prior work [30, 49], designed to maximize the number of reports collected while minimizing sleep disturbance.

First, up to 10 “sleep onset” dream reports were collected during the first hour of the night, following 30, 60 or 90 seconds of PSG-defined sleep (latencies following a randomized order). The use of numerous sleep onset awakenings early in the night allows dreams to be collected rapid succession, yielding a larger amount of data from each participant. This technique has been used successfully in a number of our past studies [30, 50], and importantly, these sleep onset reports fall squarely under our definition of "dreaming” as *the subjective experience of mental activity occurring during sleep*. Offline sleep scoring determined that the majority of these sleep onset awakenings were made during N1 sleep (N = 255, 81.7%), with an additional N = 45 (14.4%) from N2, and N = 12 (3.8%) during wake. Reports scored as being from wake were excluded from further analysis.

Three additional reports were collected later the night: one from Stage 2 NREM sleep, one from REM (rapid eye movement) sleep, and a final report upon morning awakening, regardless of sleep stage. Stage 2 NREM reports were elicited after at least 10 continuous minutes of Stage 2 sleep, and REM reports were elicited after at least 5 continuous minutes of REM sleep. These reports were separated from the sleep onset reports by at least 1hr and from each other by at least 30 minutes. Order of the Stage 2 NREM and REM awakenings was counterbalanced across participants. The morning awakening report was scheduled for ≈8:00am (8hrs following the completion of the 1hr sleep onset report collection period), regardless of sleep stage. At each of these time points, participants were awakened by calling their name, and instructed to verbally report “*everything that was going through your mind*” just before they were called. All reports were digitally recorded, and later transcribed.

One variable of interest was the time of night at which each report was collected, quantified as minutes elapsed since sleep onset. However, time of report collection was very unevenly distributed across the night and could not be treated as a normally-distributed continuous variable. Therefore, in the below-reported analyses, we categorized reports by quartile of the night, rather than treating time of night as a continuous variable.

#### Participant identification of past and future waking sources

The following morning, participants listened to the audio recording of each of their reports from the previous night, in the order that they were collected. For each report, participants were guided in completing a questionnaire asking them to identify potential *past* and *future* sources that they believed influenced their experience. Specifically, participants were asked “*is this report related to*” the following categories of experience (see Table 1 for example reports):

*Specific past episode*. Participants were instructed to respond in the affirmative to this category if they felt that the dream was caused by a *specific experience that occurred at a particular time and place in the past* (an episodic memory). If they responded in the affirmative, participants were asked to describe what the event was, and to indicate when it occurred (forced choice between *Yesterday/Within the Last Week/Within the Last Month/Within the Last Year/More than 1 Year Ago)*.

*Specific anticipated future episode*. Participants were instructed to respond in the affirmative to this category if they felt that the dream was caused by a *specific experience that will occur at a particular time and place in the future* (a future episode). If they responded in the affirmative, participants were asked to describe what the event is, and to indicate when they anticipate that it will occur (forced choice between *Later Today*, *Within the Next Week*, *Within the Next Month/Within the Next Year/More than 1 Year from Now)*.

**Table 1:** Example past and future episodic sources.

Content Type	Temporal Orientation of Source		
	Past	Future	Past + Future
Dream Report	“I was just seeing the inside of a house and some people, one of whom might have been *Jennifer Aniston*.*”*	“There was some sort of musical scale going up… And I think I was dreaming about Dr. [X], my choral conductor…*I was watching the guys in Furman Singers sing a song about the Air Force in McAllister [auditorium]*. But it was kind of like I was watching it from above, like on the catwalk or something.”	“I was thinking about *‘Little House on the Prairie’*… I was dreaming that, like, one of the main characters, *they had to move back*, and they had a little wagon and they had to go back to their old house.”
Waking Source	Jennifer Aniston “was mentioned in an entertainment news story I saw earlier in the day”	“The Furman Singers will sing at the band concert on Tuesday”	The participant reported that the dream was caused by watching “*Little House on the Prairie*” in the past, but also by the fact that their family would be moving to Oregon in the near future

*Note*. Examples of participant-identified past and future episodic memory sources. Participants were instructed to distinguish specific episodes (defined as particular past or future events that occurred/will occur at a specific time and place) from general categories of past experience or future concerns.

Even when participants cannot link their dream to a specific spatiotemporal event, they often identify dreams as related to general categories of past or future experience. Therefore, the questionnaire additionally allowed participants to indicate that their experience was related to:

*General category of past events*. Participants were instructed to respond in the affirmative to this category if they felt that the dream was caused by *a general category of past experience*, but not by any specific event that occurred at a particular time and place (not an episodic memory, potentially a semantic memory).

*General category of future concerns*. Participants were instructed to respond in the affirmative to this category if they felt that the dream report was caused by *a general category of future concerns*, but not a specific experience that will occur at a particular time and place in the future (not a future episode).

Dream reports could be classified as relating to multiple types of waking sources, and could be classified as related to multiple sources within a single waking source type. Participants were only asked to provide detail on what the waking event was and when it occurred for episodic-type sources. For all identified waking sources, the participant used a 7-point Likert scale to rate their confidence in the association between the dream report and its putative source.

#### Statistical analysis methods

Statistical analyses were conducted in R [51]. Many observations reported here are descriptive estimates of proportions. We estimated 95% binomial confidence intervals using the Clopper–Pearson “exact” method, as implemented in binom package for R (Dorai-Raj, 2014). Where statistical comparisons were conducted, mixed effect models were used to account for the multilevel nature of these data, in which each participant contributes multiple dream reports. Specifically, mixed effect models were conducted with observations grouped by participant, and intercepts were allowed to vary by participant. Where outcome variables were categorical, mixed effect logistic regression models were implemented using the generalized linear model function ‘glmer’, in the lme4 package [52]. Here, statistical significance was assessed using Wald chi-square tests, and pairwise contrasts were conducted using emmeans package for R [53]. Where outcome variables were continuous, mixed effect models were conducted using the lme4 and lmerTest packages for R [54]. ANOVA and pairwise test statistics derived from these models used Satterthwaite’s method of estimating degrees of freedom.

## Results

In total, N = 469 dream reports were collected from 48 participants. Participants contributed an average of 9.77±3.24SD reports (range: 4–13). N = 374 reports contained at least some mental content (79.74%, 95% CI [75.82%, 83.29%]), with the remaining 95 consisting of a failure to recall any experience (e.g. “I don’t know”/”I can’t remember”; 20.26%, 95% CI [16.71%, 24.18%]). Of reports with content, 272 (72.73%) were from sleep onset awakenings, 29 (7.75%) from NREM sleep, 38 (10.16%) from REM sleep, and 35 (9.36%) were morning-collected reports. Among content-filled morning-collected reports, 18 (51.4%, 95% CI [40.0%, 68.6%]) were reported from N2 sleep, 16 (45.7%, 95% CI [28.8%, 63.4%]) from REM sleep, and 1 (2.9%, 95% CI [0.0%, 15.0%]) from N1 sleep. Despite the repeated awakening protocol, participants were able to obtain an average of 468.0±77.3 SD min of sleep during the night, including 28.4±11.8SD min of stage N1, 234.2±75.9 min of stage N2, 119.7±29.0 min of stage N3 and 85.7±28.2 min of REM sleep.

### Prevalence of past and future waking sources

**Fig 1:**
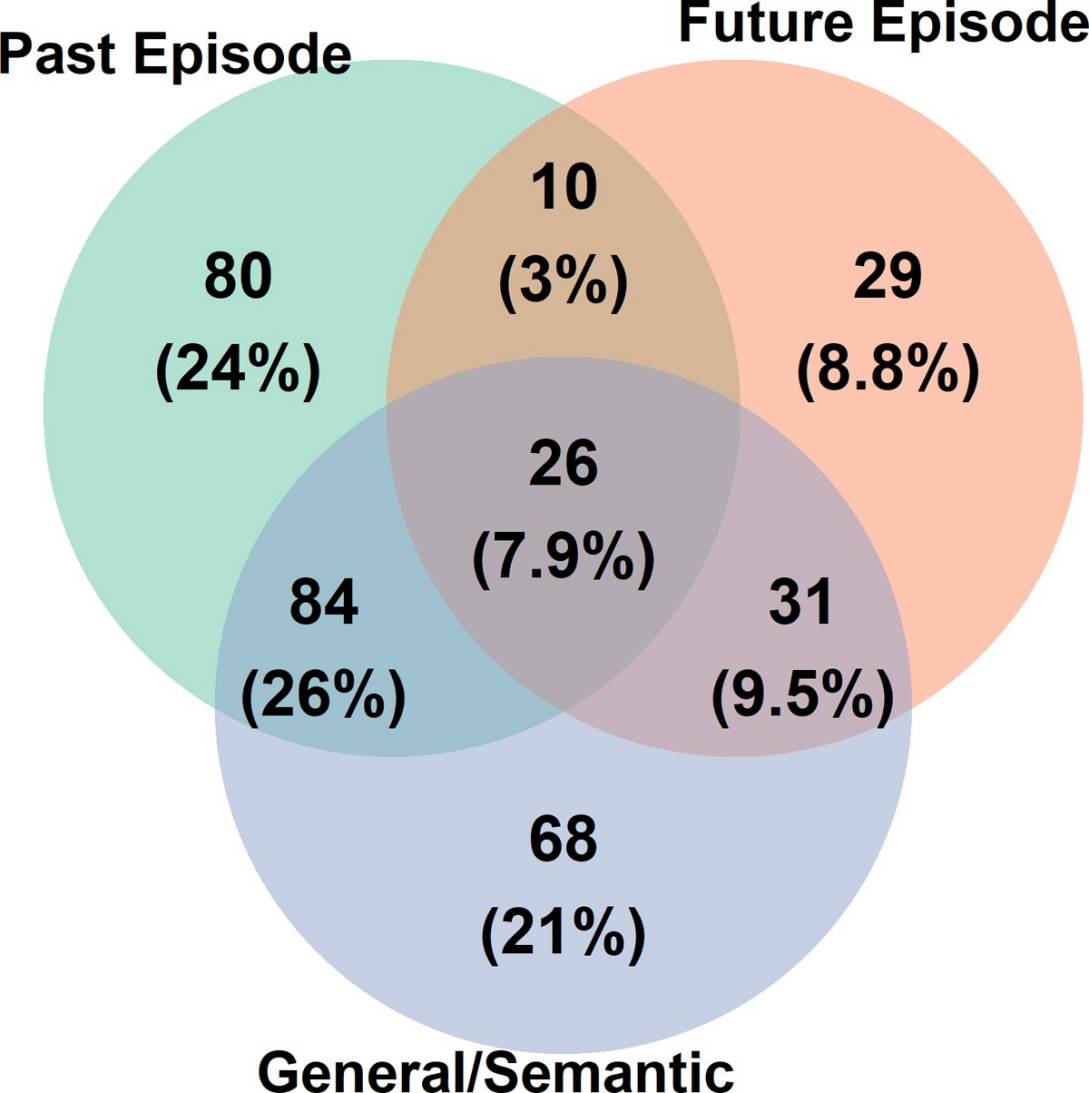
Co-occurrence of past and future dream sources. Data are shown as the raw number of reports identified in each category, and as a % of all dreams with a waking source identification. The “general/semantic” category collapses across reports identified as related to general categories of past experience and future concerns.

Of reports with content, 328 were identified as having at least one waking source (87.7%, 95% CI [83.94, 90.85]). 200 were identified as related to a specific event in the past (53.48%, 95% CI [48.28%, 58.62%]), 96 as related to a specific anticipated event in the future (25.67%, 95% CI [21.32%, 30.41%]), 164 as related to a general category of past events (43.85%, 95% CI [38.75%, 49.04%]), and 78 as related to a general category of future concerns (20.86%, 95% CI [16.85%, 25.33%]). As summarized in Fig 1 and Table 3, nearly half of reports with a waking source were traced to multiple different sources (164 reports or 50%, 95% CI [44.46%, 55.54%]). Of special relevance to our hypotheses, many dreams traced to an impending future episode were also judged to be related to one or more specific past episodic memories (n = 36, or 37.5% of all reports with a future episodic source, 95% CI [27.82%, 47.97%]).

**Fig 2:**
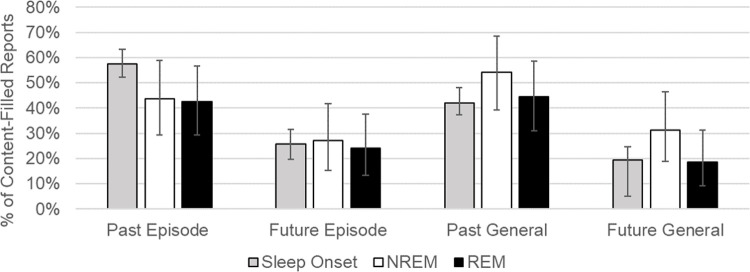
Dream sources by sleep stage. Proportion of content-filled dream reports from each sleep stage by type of waking source. REM = Rapid eye movement sleep. NREM = Non-rapid eye movement sleep. Error bars = 95% CI. Binomial confidence intervals calculated using the Clopper–Pearson “exact” method.

Dreams were more likely to be associated with past, relative to future episodes (Wald χ2(1, *N* = 689) = 27.47, p<0.00001; Fig 2). Similarly, dreams were more often associated with general past experiences than with general future concerns (Wald χ2(1, *N* = 688) = 16.61, p = 0.00005; Fig 2).

Episodic sources were unevenly distributed across time, with events in close temporal proximity to the experimental night most commonly identified as a dream source. As described in Table 2, this was the case for both past and future sources. Thus, dreams were most often identified with events that occurred yesterday or that will occur tomorrow (37%, 95% CI [30.3%, 44.09%] and 23.96%, 95% CI [15.83%, 44.09%] of identified sources, respectively).

**Table 2:** Temporal origin of episodic sources in content-filled reports.

Distance from Present	Past Sources				Future Sources			
	*n*	%	95% CI		*n*	%	95% CI	
<1 Day	74	37.0%	[30.3%,	44.0%]	23	24.0%	[15.8%,	34.0%]
Within a week	33*	16.0%	[11.6%,	22.0%]	16	16.7%	[9.8%,	26.0%]
Within a month	20*	10.0%	[6.2%,	15.0%]	13	13.5%	[7.4%,	22.0%]
Within a year	24*	12.0%	[7.8%,	17.0%]	13	13.5%	[7.4%,	22.0%]
>1 Year	27*	14.0%	[9.1%,	19.0%]	5*§	5.2%	[1.7%,	12.0%]
Unspecified^⸸^	22	11.0%			26	27.1%		
Total	200				96			

*Note*. Percentages for past and future sources are relative to the total 200 reports identified as related to a past episode and 96 reports related to a future episode, respectively.

* = category was reported significantly less frequently than “<1 Day”;

§ = category was reported significantly less frequently than “Within a week”. Chi-square tests, p<0.05 significance threshold.

^⸸^Participant did not answer this question or indicated uncertainty about the timing of the event.

### Future-oriented dreams are proportionally more common later in the night

**Fig 3:**
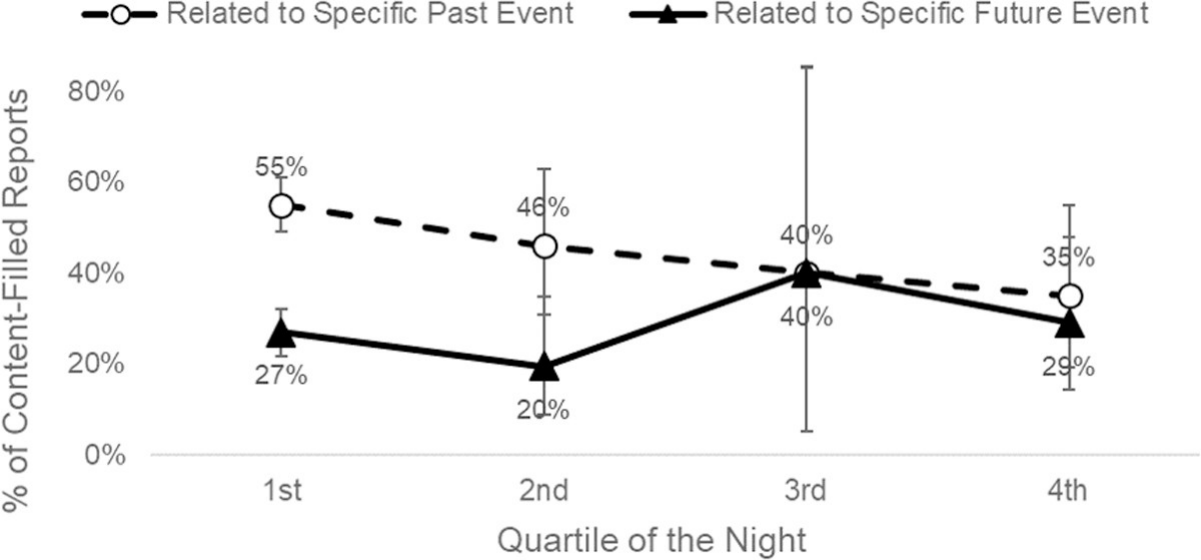
Temporal orientation of episodic sources varies by time of night. Proportion of content-filled dream reports related to past and future episodes, by quartile of the night. Error bars = 95% CI. Binomial confidence intervals calculated using the Clopper–Pearson “exact” method.

Future-oriented dreams became proportionally more common later in the night. The probability that a dream would be linked to a specific past episode declined across the night, such that dreams reported in the 4^th^ quartile of the night were less likely relate to a past episode than those in the 1^st^ quartile (p = 0.03). In contrast, the probability that a dream would be associated with an impending future event remained stable across the night, with no difference between the 1^st^ and 4^th^ quartiles (p = 0.79). Thus, as displayed in Fig 3, dreams in the 1st quartile of the night were more than twice as likely to be linked to a past, rather than a future event (55.16%, 95% CI [49.14%, 61.07%] of reports vs %, 26.69%, 95% CI [21.61%, 32.27%] of reports). In contrast, dreams in the final quartile of the night showed similar rates of past vs future event incorporation (35.48%, 95% CI [19.23%, 54.63%] vs 29.03%, 95% CI [14.22%, 48.04%] of reports). However, the interaction between quartile (1st vs 4th) and temporal orientation (past vs future) did not reach statistical significance (Wald χ2(1, *N* = 585) = 2.98, p = 0.08).

The probability that a dream would be linked to a general category of past events did not vary by time of night (1^st^ vs 4^th^ quartile: p = 0.97), nor did the probability that a dream would be linked to a general future concern (1^st^ vs 4^th^ quartile: p = 0.14). There was also no interaction between quartile (1st vs 4th) and temporal orientation (past vs future) for general categories of past and future events (Wald χ2(1, *N* = 584) = 1.00, p = 0.32).

### Effect of sleep onset report latency (30s vs 60s vs 90s)

At sleep onset, we examined whether dream memory sources were affected by how long participants had been asleep prior to awakening (30s vs 60s vs 90s awakening latencies, see Methods). Participants were less likely to report dreams relating to general future concerns from longer-duration awakening latencies (Wald χ2(1, *N* = 247) = 6.24, p = 0.04). Specifically, reports following 90s of sleep were less likely to reference a future concern, relative to those reported following either 60s (p = 0.02) or 30s of sleep (p = 0.03). Awakening latency did not significantly affect the incorporation of anticipated future episodes (Wald χ2(1, *N* = 247) = 4.55, p = 0.10), nor the incorporation of past memory sources (episodic sources: Wald χ2(1, *N* = 248) = 4.77, p = 0.09; general sources: Wald χ2(1, *N* = 247) = 0.76, p = 0.68).

#### Effects of sleep stage on dream sources

**Fig 4:**
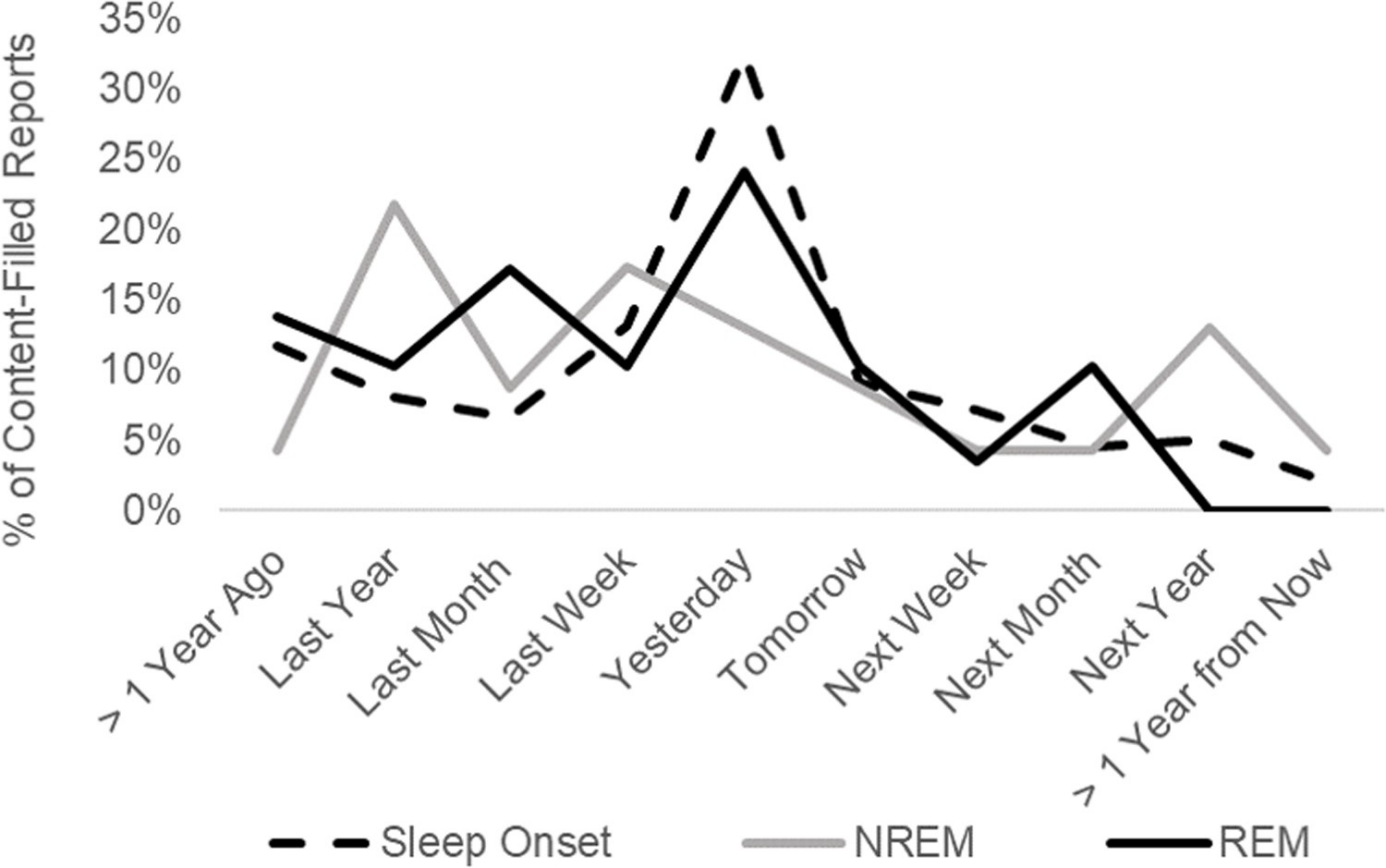
Past and future temporal references by sleep stage. Episodic sources by temporal origin and sleep stage. Percentages are relative to all content-filled reports with a valid source. NREM = Non-rapid eye movement sleep. REM = Rapid eye movement sleep.

Identification of past episodic memory sources varied significantly by sleep stage (Wald χ2(1, *N* = 345) = 6.17, p = 0.046; see Fig 2), with past episodic sources incorporated into sleep onset dreams marginally more frequently than either REM (p = 0.06) or NREM dreams (p = 0.06). There was no significant effect of sleep stage on the frequency with which anticipated future episodes, general categories of past events, or general categories of future concerns were identified as dream sources (all Wald test p-values >0.12). As illustrated in Fig 4, temporal distance of episodic sources from the present was approximately equivalent across sleep stages. Sleep stage did not affect the temporal distance of episodic sources for either past (F(2,155) = 0.59, p = 0.55) or future sources (F(2,59) = 1.00, p = 0.37).

### Waking source combinations

#### Co-occurrence of past and future episodic sources

In n = 36 reports, participants identified a single dream as related to both a specific past event and a specific anticipated future event (Table 3 and Fig 1). In some cases, participants identified distinct past and future sources that were semantically related to each other. For example, one participant reported:

*I was thinking about ‘Little House on the Prairie’. It’s this TV show about a farmer and I was dreaming that like one of the main characters, they had to move back, and they had a little wagon and they had to go back to their old house.*

The participant associated this dream both with watching *“Little House on the Prairie”* more than a year ago, and with the fact that their family would be moving to Oregon the next month.

**Table 3:** Co-occurrence of waking sources by category.

Primary Source	Secondary Source(s)															
	Past Episode				Future Episode				General Past				General Future			
	*N*	*%*	*95% CI*		*N*	*%*	*95% CI*		*N*	*%*	*95% CI*		*N*	*%*	*95% CI*	
Past Episode	19	9.5%	[5.8%	14.4%]	36	18.0%	[12.9%	24.0%]	99	49.5%	[42.4%	56.6%]	33	16.5%	[11.6%	22.4%]
Future Episode	36	37.5%	[27.8%	48.0%]	5	5.2%	[1.7%	11.7%]	29	30.2%	[21.3%	40.4%]	44	45.8%	[35.6%	56.3%]
General Past	99	60.4%	[52.4%	67.9%]	29	17.7%	[12.2%	24.4%]	0	0.0%	[0.0%	2.2%]	33	20.1%	[14.3%	27.1%]
General Future	33	42.3%	[31.2%	54.0%]	44	56.4%	[44.7%	67.6%]	33	42.3%	[31.2%	54.0%]	0	0.0%	[0.0%	4.6%]

*Note*. For dreams containing each category of waking source, the number and % of dreams that were additionally traced to a second source in the same or a different category. There were a total of 200 reports with at least one past episodic source, 96 reports with at least one future episodic source, 164 reports with at least one general past source, and 78 reports with at least one general future source

In other cases, reports were traced to distinct, apparently unrelated past and future sources. For example, one participant reported the following dream, associating separate segments of the dream with having gone running on the previous day, and with a bake sale planned for the following week:

*… I was running on the Swamp Rabbit [a local biking/jogging trail]…Oh, I was thinking about my job. I work at Kinetics two days a week, and we’re doing a bake sale.*

### Co-occurrence of multiple past or multiple future sources

In some cases, multiple different episodic memories of the same type were identified as the source of a dream. 19 dreams were associated with multiple past episodes (9.2% of all dreams associated with a past episode, 95% CI [5.6%, 14.0%]), and 5 dreams were associated with multiple different future episodes (5.1% of all dreams associated with a future episode, 95% CI [1.7%, 11.4%]). For example, one participant reported this dream that combined two seemingly unrelated recent past episodes:

*I was just seeing the inside of a house and some people, one of whom might have been Jennifer Aniston and umm, there was a couch I think there was some kind of fragrance plug-in thing like a wallflower from Bath and Body Works and somebody was going to look at it.*

The participant indicated that Jennifer Aniston *“was mentioned in an entertainment news story I saw earlier in the day”*, and also that the plug-in fragrance dispenser was something that they had been “*thinking about buying … and went to look at them the other day”*.

As shown in Table 4, of the n = 19 dreams traced to multiple past episodes, 8 combined an episodic memory from the previous day with a more remote memory from the previous week (n = 3), month (n = 2), or years (n = 3). In some cases, the recent episodic memory was strongly related to the co-occurring remote memory, with an experience from the previous day seeming to trigger a dream about a related remote memory. For example, one participant reported that:

*It was sort of like I was back at Disney for [study away] … nothing weird or anything, completely normal yeah we were just walking around in the park, but it was at Furman which doesn’t make sense.*

The participant indicated that this dream was caused by seeing friends that had been on this Disney study away trip yesterday, and by the actual experience of studying away at Disney during the previous year. Another n = 9 reports instead combined multiple different remote past episodes, but only a single dream was identified with multiple different episodes from the previous day (Table 4).

**Table 4:** Combinations of episodic sources from different timepoints.

Source Type	Type of Temporal Source Combination											
	Proximal + Proximal				Remote + Remote				Proximal + Remote			
	*n*	*%*	*95% CI*		*n*	*%*	*95% CI*		*n*	*%*	*95% CI*	
Future Episodes	0	0.0%	[0.0%	52.2%]	3	60%	[14.7%	94.7%]	2	40%	[5.3%	85.3%]
Past Episodes	1	5.3%	[0.1%	26.0%]	9	47.4%	[24.4%	71.1%]	8	42.1%	[20.3%	66.5%]
Total (Future +Past)	1	4.2%	[0.1%	21.1%]	12	50.0%	[29.1%	70.9%]	10	41.7%	[22.1%	63.4%]

*Note*. Count and % of memory source combinations in each of 3 categories. *Proximal* = Memories from the previous week or events expected to occur in the next week. *Remote* = Memories from >1 week ago or events expected to occur >1 week in the future. In one case, the participant reported multiple past episodic sources for a dream but did not indicate the time at which the episodes occurred.

#### Future event combinations

Of the 5 dreams traced to multiple future episodes, n = 2 combined an event that would occur the next day with an event that would occur one or more years in the future, with the remaining 3 combining multiple events that were anticipated > 1 month in the future.

## Discussion

Dreams have long been known to reflect the reactivation of past memory [31]. Yet the factors controlling *which* specific memory fragments appear in dreaming and *why* have remained obscure. Our current observations suggest that constructive episodic stimulation, a proposed framework for understanding prospective waking thought, may be useful in explaining the construction of dream cognition. We observed that >25% of dreams were identified as related to specific impending future events. Yet few were a “realistic” simulation of any single past or future episode. Instead, participants reported dreams that intermingled elements drawn from multiple past and future sources; Over a third of dreams about a future event additionally incorporated elements of one or more specific past episodic memories. This suggests that dreams leverage fragments of past memory in constructing imagined scenarios that anticipate future events, much as described in the case of waking cognition [2, 3, 6]. Potentially, this could reflect a function for dreaming, in which past memory stores serve as the raw material for rehearsing possible futures. Thus, the participant who dreamed of *“Little House on the Prairie”* reported that this dream anticipated her family’s impending move to Oregon. Perhaps, with little real-life experience to draw on in simulating what this move might look like, the dream is instead constructed from loosely associated imagery from a fictional television show.

We also observed a trend for future-oriented dreams to become proportionally more common later in the night, as participants move toward morning waking. This echoes a small-sample study by Malinowski & Horton [55], which observed a non-significant but substantially sized increase in participant-rated similarity of dreams to future events across the night. In a subsequent study, the same authors also reported that late in the night, participant ratings of recent-memory incorporation were correlated with their ratings of future-event incorporation [56]. Just as temporal proximity to the past may explain the well-known recency bias in dream memory sources [57], temporal proximity to impending future events of the following days may drive future sources to become relatively more common in dreams reported later in the night.

In apparent contradiction with the present observations, Speth et al. [58, 59] reported that participants were very unlikely to describe anticipation of the future in dream reports, relative to in reports of waking thought. However, unlike in the present study, Speth et al. assessed future-thinking by coding explicit language specifically mentioning the future within the text of the dream report itself (e.g. “*I’m thinking about the formal tomorrow night*” [58]). This method would not have been sensitive to instances in which participants dreamed of imagined scenarios that related to the future, without specifically remarking on this in the text of the dream report (which they were not instructed to do). In the above example, for instance, if the participant dreamed of attending a formal it would not have been scored as a future-related dream unless the participant happened to provide an unsolicited comment that they would, in real life, be attending a formal the following night. We found that participants very often described dreams as relating to the future when prompted to comment on this in the morning session, even when this was not apparent in the text of the dream reported the night before.

Even when no future source was present, dreams often combined multiple past sources. Over half of dreams traced to a past episodic source were also identified with at least one other past episodic or general/semantic memory. Co-activation of multiple past memories may itself serve a memory processing function. When a recent episode from the previous day is co-activated with related remote or semantic memories, synaptic connections between the recent memory and its neocortical associates may be strengthened, supporting the gradual integration of new information with existing knowledge structures. Indeed, a handful of studies suggest that sleep facilitates memory integration [44, 45]. McClelland, McNaughton & O’Reilly first speculated this decades ago, proposing that sleep may “permit both new and old information to be played back in closely interleaved fashion” [60].

Yet despite the resemblance of dreams to waking constructive episodic simulation, dreaming is likely not strictly *the same* process as waking prospective thought. First, future-related dreams are often highly implausible scenarios that would be unlikely or impossible to actually occur in waking life. In part, this might be attributed to weak associative strengths between the memory elements appearing in the dream and the future event that the dream seems to anticipate. For example, a participant in one prior study anticipated being retested on a computerized virtual maze task the next morning, dreaming of being lost in a coliseum searching for prize [37]. The dream was described as reminiscent of the virtual maze task, but its specific elements were highly dissimilar to it, as the maze included neither any human characters nor any imagery of a coliseum. In this sense, future-oriented dreams might be described as less “realistic” than those present in goal-directed forms of waking prospective thought. But this does not imply that such dreams are non-functional. To the contrary, the activation of weak associations may be a critical component of creative, flexible, and divergent thinking [61–63]. Prospective cognition during sleep also differs from that during wake in that some brain regions supporting episodic future stimulation in wake are conspicuously *deactivated* during sleep, including dorsolateral prefrontal cortex and regions of the posterior parietal lobes [64–66]. Deactivation of these regions, both associated with cognitive control during wakefulness, could reflect a loss of top-down constraint during the construction of prospective dreams, as opposed to waking thought.

### Relation to simulation-based theories of dreaming

This paper is not unique in describing dreaming as a “simulation”. Indeed, cognitive theorists have described dreaming as a form of reality simulation for decades [67–72]. Influential simulation-based accounts of dreaming have included the “threat simulation” theory proposed by Antti Revonsuo [73], a related characterization of dreams as “social simulation” [72], and Domhoff’s neurocognitive theory of dreaming, which emphasizes the unique embodied nature of dream simulations [68, 74]. According to all of these accounts, dreams are notable for being a relatively realistic analogue to waking perception, and are in many ways continuous with waking life, for example incorporating themes, characters, concerns and memories from waking experience. Threat simulation theory proposes that dreaming specifically evolved as an adaptive system for simulating dangerous events, allowing our ancestors to enhance their preparedness for future life-threatening situations through offline mental rehearsal in a realistic virtual environment. Similarly, social simulation theory proposes that dreaming specifically evolved as an adaptive system for simulating social interactions. Domhoff’s description of dreaming as “embodied simulation” differs, in that it does not presume that dreams simulate only particular, restricted categories of waking life experience, and does not assert that dream simulations function to prepare us for the future [67, 68].

The current observations are at least partially compatible with all of these views, yet the notion of dreaming as a “constructive episodic simulation” differs in key respects. First, unlike the threat and social simulation theories, we do not presume that dreams evolved to allow us to selectively rehearse a particular category of future event. Second, while all of these theories presume that dreams draw on past memory, they do not emphasize the specific process by which simulations are constructed from memory. The current study presents novel evidence on this point, demonstrating how the simultaneous reactivation of multiple memory fragments can result in novel dream scenarios relevant to an individual’s personal future. This core insight of the constructive episodic simulation hypothesis is not incompatible with prior theories of dream simulation, but empirical evidence describing its occurrence has been limited. Third, our observations are unique in suggesting that reactivation of past memory fragments can be driven by knowledge of a *specific* event that is going to occur at a known time in the near future. In contrast, both the threat and social simulation theories view dreaming as a platform to enhance more general preparedness for future threatening or social situations, but do not discuss dreaming as a result of the knowledge of specific impending future events.

Finally, threat simulation and social simulation theory both propose that the phenomenological content of the dream is itself functional, providing a realistic rehearsal that conferred a survival advantage to our ancestors and was specifically selected for during evolution. In contrast, we do not presume that the experiential component of dream is itself functional. The simulations seen in dreaming are often highly unrealistic and may be better conceptualized as *reflecting* a functional brain process (the reactivation and continued consolidation of future-relevant memories) rather than themselves comprising an evolutionarily advantageous realistic rehearsal opportunity.

### Limitations

In identifying the potential memory sources of their own dream reports, participants have the advantage of a greater knowledge of their own past memories and future concerns than can be gleaned by independent judges. As a result, participant-ratings of dream sources are more sensitive, better able to capture remote past and mundane, seemingly insignificant details as a source of dream content [75]. At the same time, this method comes with some inherent limitations.

First, participants are typically unfamiliar with the concept of episodic memory. Although participants were specifically instructed that past/future episodes are *specific events that occur at a particular time and place*, it was not always clear whether reported episodic sources adhered to this standard. For example, in the *“Little House on the Prairie*” dream described in the main text, a participant identified the television show “*Little House on the Prairie*” as an episodic memory source. However, because they did not include details establishing that the source was a specific episode of the show seen at a specific time, it was not clear whether this source was truly episodic, or might be better classified as a semantic memory. In this respect, the line between episodic and semantic memories may be somewhat blurred in this study.

Second, probing participants to identify dream sources may introduce demand characteristics. Participants were not aware of the specific hypotheses of the current study, and there is no evidence that participants fabricated any responses. However, because there is no way to independently verify the accuracy of participants’ subjective report, we cannot rule out the possibility that demand characteristics inflated waking source identification rates.

Third, this study cannot distinguish whether dreams identified as “about the future” are caused purely by a participant’s knowledge of an upcoming event, or alternatively, by a particular past episode in which the participant talked about, learned about, or thought about the upcoming event. In in either case, future-oriented dreams must certainly have been caused by some past experience or cognition. In this sense, dreams “about the future” are necessarily also dreams about the past.

Finally, a very large portion of dreams collected in this study were from short-latency sleep onset awakenings. Because of this, our observations could be skewed to reflect peculiarities of this particular type of mentation [76], rather than being representative of dreams from all sleep stages at all times of night. In particular, only 10% of the dreams described here were collected from REM sleep, and we may have been underpowered to detect sleep-stage differences in dream content. Thus, despite the null sleep stage effects reported here, the effect of sleep stage on past and future episode incorporation could yet be a fruitful area for continued research.

## Conclusions

These observations suggest that dreams combine fragments of past memory into novel constructions, and that these novel constructions often relate to an anticipated future event. In contrast to waking prospective cognition, during sleep, this is unlikely to be a goal-driven process under top-down control. Instead, prospective dreams may be an emergent phenomenon occurring when various fragments of future-relevant episodic and semantic memory are co-activated and combined in novel ways. The result is a dream that participants perceive to be “about the future”. Despite the fact that such dreams are often bizarre and unrealistic, this offline reactivation of future-relevant past memory could potentially function to help prepare us for the future.

## Supporting information

S1 File Supplementary information. Supplementary methods and results.
(DOCX)
