# Supplementary material for: Constructive episodic simulation in dreams
Source: PLoS One. 2022 Mar 22;17(3):e0264574. doi: 10.1371/journal.pone.0264574 (PMC8939783; doi:10.1371/journal.pone.0264574)
Supplement: S1 File — Supplementary methods and results. (DOCX) [file pone.0264574.s001.docx]

**Supplementary Information: Episodic Future Simulation in Dreams**

Erin J. Wamsley

*Department of Psychology and Program in Neuroscience*

*Furman University, Greenville SC*

**Supplementary Methods**

**Sample Size and Power**

The current sample far exceeds the size of that reported in typical studies of dream content, both in terms of the number of participants and the number of dream reports. Although some hypotheses tested here were novel, with no prior literature available for effect size estimation, there were two areas in which we were able to project expected effect sizes based on the prior literature:

a) Prior studies of how time of night affects dream content have consistently reported large effect sizes (Malinowski & Horton, 2014; Nielsen, 2004; Wamsley et al., 2007). Most similar to the current project is a small-sample study (N=49 dreams from 16 participants) reporting a large increase in participant-rated similarity of dreams to future events between the 1^st^ and 4^th^ quartiles of the night (Cohen’s d=1.1). With nearly 4x the number of observations (N=481 dream reports from 48 participants), the current study was powered at >0.99 to detect a similarly large effect size (Cramer’s V=0.5) in a chi-square test, and was powered at >0.8 to detect an effect as small as Cramer’s V=0.16.

b) Past studies of sleep stage differences in episodic memory incorporation have reported smaller effect sizes. In a meta-analysis combining data across three prior investigations (with a combined total of N=110 dream reports), Baylor & Cavallero (2001) reported an effect of Cohen’s d=0.19 for the greater incorporation of episodic memory into NREM, as compared to REM dreams. The current study would be powered at 0.78 to detect a similarly small effect (e.g. Cramer’s V=0.15) in a chi-square test of independence comparing probability of past or future episode incorporation between REM and NREM dreams. Thus, the current study may have been less well-powered to detect sleep stage differences in past and future episode incorporation.

Effect size and power calculations were conducted in R, using the powerAnalysis package (Felix Yanhui Fan (2017). powerAnalysis: Power Analysis in Experimental Design. R package version 0.2.1. https://CRAN.R-project.org/package=powerAnalysis)

**Missing data**

*Missing Dream Reports*

There were 24 audio files from 7 participants that were either not sufficiently audible to the transcriber or were lost or corrupted following data collection. Because these crucial source data were not available, waking source questionnaire data for these reports were not included in any of the analyses.

*Missing Questionnaire Responses*

There were 2 cases in which, although judges classified a collected report as content-filled, the participant failed to compete a memory questionnaire about it. These reports are included in the count of collected and content-filled reports, but excluded from all analyses of memory sources. In one additional case, a participant only partially completed the waking source questionnaire, failing to respond to questions about past general, future episodic, or future general sources. This report is selectively excluded from analyses for which data were missing. There were n=49 reports for which a participant indicated that a dream had a specific past or future episodic source, but then either did not complete the question regarding the specific timepoint at which this episode did/will occur, or wrote comments indicating uncertainty about the timing. Analyses of the temporal origin of episodes therefore only include data from the n=256 reports with valid data for this question.

*Missing Sleep State or Report Timing Data*

There were N=39 awakenings for which the PSG-defined sleep stage at the moment of awakening could not be accurately determined, due to pervasive EEG artifact or failure to accurately record a marker on the PSG record. In the N=22 (4.6%) cases where a marker was not accurately recorded, we also were additionally unable to accurately determine of the clock time of the awakening. Reports with missing sleep stage or timing data are selectively excluded from analyses of these particular variables.

**Supplementary Results**

**Confidence Ratings**

Participants’ confidence in their memory source identifications was generally high, with >50% of identifications rated as a 6 or 7 on a 7-point scale for all waking source types. Confidence ratings did not differ significantly between waking source types.

**Supplementary References**

Baylor, G. W., & Cavallero, C. (2001). Memory sources associated with REM and NREM dream reports throughout the night: A new look at the data. *Sleep*, *24*(2), 165–170.

Malinowski, J. E., & Horton, C. L. (2014). The effect of time of night on wake–dream continuity. *Dreaming*, *24*(4), 253–269. https://doi.org/10.1037/a0037817

Nielsen, T. A. (2004). Chronobiological features of dream production. *Sleep Medicine Reviews*, *8*(5), 403–424.

Wamsley, E. J., Hirota, Y., Tucker, M. A., Smith, M. R., & Antrobus, J. S. (2007). Circadian and ultradian influences on dreaming: A dual rhythm model. *Brain Research Bulletin*, *71*(4), 347–354. https://doi.org/10.1016/j.brainresbull.2006.09.021
